# Radiographic determination of the tracheal indices at caudal cervical, thoracic-inlet, and intra-thoracic trachea in non-bulldog brachycephalic breeds without evidence of cardiorespiratory disease

**DOI:** 10.1186/s12917-023-03730-0

**Published:** 2023-10-02

**Authors:** Ayman A. Mostafa, Clifford R. Berry

**Affiliations:** 1 Department of Small Animal Surgery, Faculty of Veterinary Medicine, Cairo University, Giza, 12211 Egypt; 2 Diagnostic Imaging, Department of MBS, College of Veterinary Medicine, North Carolina State University, Raleigh, NC 27606 USA

**Keywords:** Radiography, Tracheal indices, Healthy, Non-bulldog brachycephalic, Dogs

## Abstract

**Background:**

Congenital tracheal hypoplasia is a component of airway syndrome affecting a wide variety of brachycephalic dog breeds. Several radiographic procedures were utilized to assess vertical tracheal diameter (VTD) in dogs. The objective of this study was to calculate the tracheal indices at the caudal cervical, thoracic-inlet, and intra-thoracic tracheal regions on the right lateral thoracic radiograph to further establish a screening protocol for diagnosis of tracheal hypoplasia in non-bulldog brachycephalic breeds. Dogs without clinical or radiographic evidence of tracheal, respiratory, or cardiovascular abnormalities were investigated. The absolute and average VTDs were normalized by manubrium length (ML), thoracic-inlet distance (Ti-D), and proximal 3rd rib width (PR3-W). Manubrium-tracheal index (M-TI), thoracic inlet-tracheal index (Ti-TI), and proximal R3-tracheal score (PR3-TS) were calculated. Correlations between averaged VTD and each of the normalizing parameters (ML, Ti-D, and PR3-W), and between M-TI and each of the previously established procedures (Ti-TI and PR3-TS) were determined.

**Results:**

Eighty healthy subjects met the inclusion criteria for the study. There were significant differences (*P* ≤ 0.0001) among the means of absolute and normalized VTDs at the 3 tracheal levels. The smallest VTD was identified at the thoracic inlet. The average tracheal diameter showed a better correlation with ML (*r*_*s*_=0.81, *P* < 0.0001) compared to Ti-D and PR3-W. There was a strong correlation (*r*_*s*_=0.83, *P* < 0.0001) between the averaged M-TI and Ti-TI.

**Conclusion:**

Radiographic M-TI could be an alternative to traditional procedures to assess the tracheal lumen in non-bulldog brachycephalic dogs. M-TI < 0.39, < 0.30, or < 0.34 at caudal cervical, thoracic inlet, or intrathoracic trachea, respectively, may indicate tracheal hypoplasia in non-bulldog brachycephalic breeds. Screening of tracheal diameter using M-TI should be recommended. However, further investigation of non-bulldog brachycephalic breeds with cardiac and/or respiratory disease is indicated.

## Background

Congenital tracheal hypoplasia is a component of obstructive airway syndrome affecting most likely brachycephalic dogs [1]. Severe tracheal hypoplasia could be a life-threatening condition that requires immediate diagnosis [2]. Many radiographic, computed tomographic, and endoscopic techniques have been established for assessing tracheal diameter and monitoring hypoplastic trachea in dogs [3–9]. In clinical practice, radiography remains the most widely utilized imaging modality to determine vertical tracheal diameter (VTD), thereby monitoring tracheal hypoplasia and selecting the proper sizes of the endotracheal tubes [7, 10, 11]. The conventional radiographic techniques relied on normalizing the thoracic inlet tracheal diameter using the corresponding thoracic inlet distance [3, 5, 6, 12, 13] or the proximal 3rd rib width [6, 13, 14]. In a recent study, manubrium length (ML) was utilized to standardize VTD measured at three different levels along the trachea (caudal cervical, thoracic inlet, and intra-thoracic) for non-brachycephalic dogs [15]. The purpose of the current study was to evaluate the manubrium tracheal index (M-TI), thoracic inlet tracheal index (Ti-TI), and proximal 3rd rib-tracheal score (PR3-TS) at the three tracheal levels for non-bulldog brachycephalic breeds. The study also aimed to calculate the correlations between the recently utilized technique (M-TI) and the conventionally established procedures (Ti-TI and PR3-TS). Our first hypothesis is that VTD would vary according to the site of the tracheal region (i.e. caudal cervical, thoracic-inlet, and intra-thoracic trachea). Our 2nd hypothesis is that the M-TI could be an alternative to the conventional Ti-TI and PR3-TS to monitor VTD in non-bulldog brachycephalic dogs. Establishing a diagnostic screening protocol for canine tracheal hypoplasia is the long-term goal of the present study.

## Methods

### Population

The study population included client-owned non-bulldog brachycephalic dogs with no history or concurrent clinical or radiographic signs of respiratory or cardiovascular disorders. Selected dogs had no record of respiratory manifestation or a heart murmur or gallop on auscultation, and their thoracic radiographs revealed no structural abnormalities concomitant with the respiratory tract or pulmonary tissue, or the corresponding cardiovascular system. Data were retrieved from July 2006 to October 2020 from the Small Animal Hospital, College of Veterinary Medicine, University of Florida. Investigated thoracic radiographic views (left lateral, right lateral, and ventrodorsal) were taken without sedation or anesthesia and at the time of the full inspiratory phase. Excluded dogs were those that revealed clinical or radiographic signs of thickened soft palate, hypoplastic or collapsed trachea, redundant tracheal membrane, or esophageal abnormalities. In addition, subjects with severe thoracic vertebral anomalies or abnormally shaped, short, or fused manubrium [15, 16] were excluded.

### Radiographic measurements

**Fig. 1:**
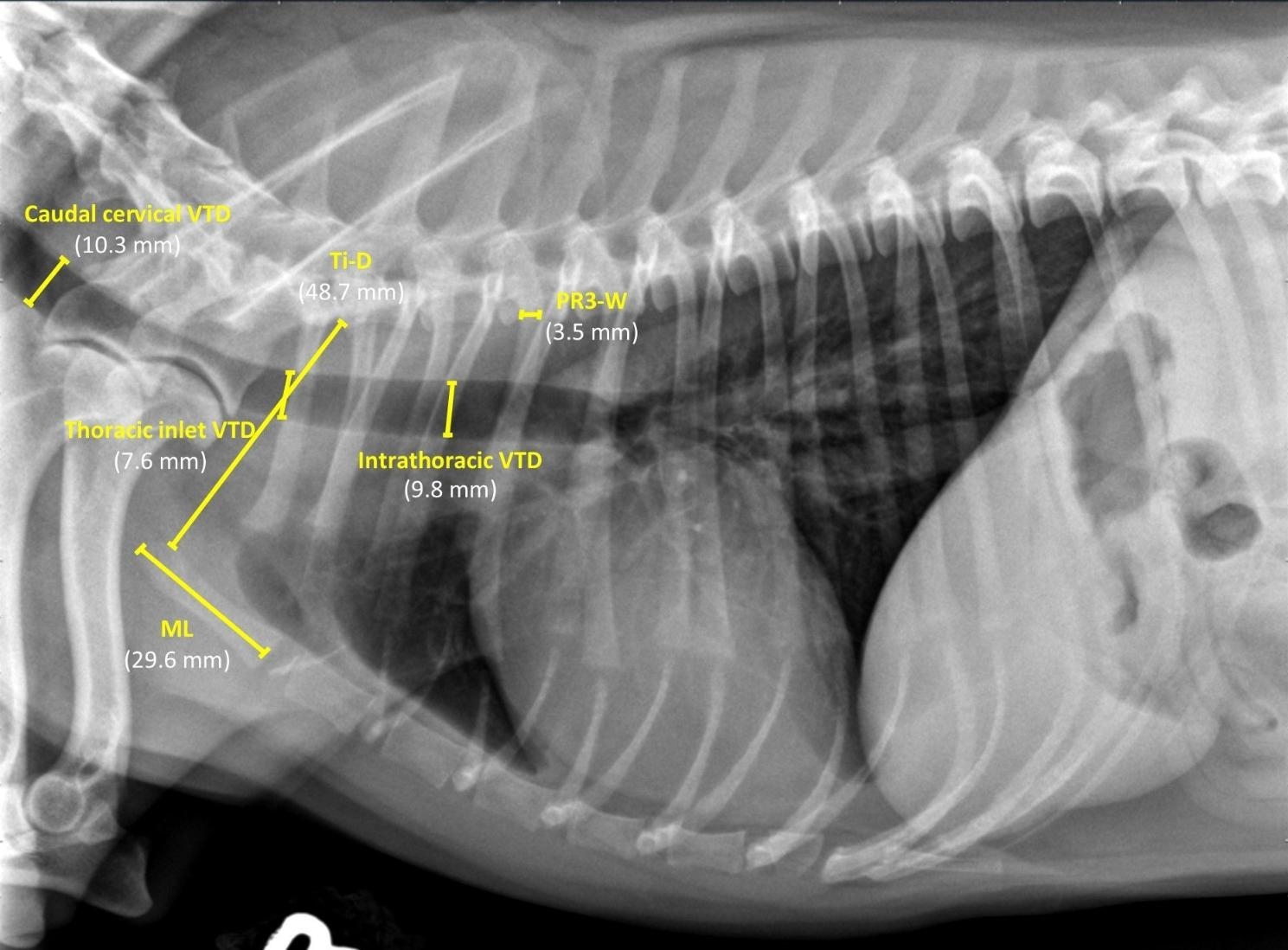
A right lateral thoracic radiograph of a healthy Shorthaired Chihuahua demonstrating the radiographic measurement of the absolute vertical tracheal diameter (VTD) at each of the caudal cervical, thoracic-inlet, and intra-thoracic tracheal regions. The figure also illustrates the radiographic measurements of the manubrium length (ML), thoracic inlet distance (Ti-D), and proximal 3rd rib-width (PR3-W) for calculating the manubrium- and thoracic inlet-tracheal indices (M-TI and Ti-TI) and proximal R3-tracheal score (PR3-TS).

The quality and positioning of all thoracic radiographs were approved by a board-certified radiologist (CRB). A single investigator (AAM) has performed all measurements on the right lateral thoracic radiographic view using the same image archiving PACs system and medical workstation (Merge PACs, Merge Healthcare Inc, Chicago, Ill). Vertical tracheal diameters were measured at the caudal cervical, thoracic-inlet, and intra-thoracic regions along the trachea (Fig. 1). The caudal cervical and thoracic-inlet tracheal diameters were measured at the levels of the middle C5 and caudal C7 vertebrae, respectively [15]. The intra-thoracic tracheal diameter was measured at the mid-way between the thoracic-inlet region and carina which is mostly located between the mid-T2 and mid-T3 vertebrae [15]. Each absolute and average tracheal diameter was normalized by the corresponding manubrium length (ML), thoracic-inlet distance (Ti-D), and proximal 3rd rib width (PR3-W) (Fig. 1) to alleviate the differences in the tracheal diameter attributed to inter-breed variation [15]. The Ti-D is the distance from the cranioventral aspect of the T1 vertebra to the craniodorsal aspect of the manubrium at its highest point (i.e., the minimum thoracic-inlet distance). The PR3-W was measured at the level of the ventral margin of the corresponding T3 vertebra [6, 14, 15]. The manubrium-tracheal index (M-TI = vertical tracheal diameter/ML), thoracic inlet-tracheal index (Ti-TI = vertical tracheal diameter/Ti-D), and proximal R3-tracheal score (PR3-TS = vertical tracheal diameter/PR3-W) were calculated at each tracheal region [15].

### Statistical analysis

Statistical analysis was performed using the commercially available GraphPad Prism software (GraphPad Prism version 8.0.0 for Windows, San Diego, California, USA). Data analysis was carried out using parametric statistical tests because variables were assumed to be normally distributed according to the central limit theorem [17]. Mean (± SD) and a 95% CI were calculated for each variable. The ANOVA and unpaired t-test were utilized to compare variables of interest, and a *P-*value < 0.05 was considered statistically significant. The correlations between the average VTD and each of the ML, Ti-D, and PR3-W were calculated using Spearman’s correlation coefficient. Moreover, the correlations between the M-TI and each of the Ti-TI and PR3-TS were determined using the same Spearman’s correlation test.

## Results

### Population

Medical records and thoracic radiographs of 88 non-bulldog brachycephalic, breeds were reviewed. Eight out of 88 dogs (9.1%) were excluded due to the presence of short (4 dogs, 4.5%), fused (3 dogs, 3.4%), and deformed (1 dog, 1.1%) manubriums. The enrolled 80 dogs met the criteria for inclusion and were admitted mostly for routine metastasis checks with no clinical or radiographic evidence of respiratory or cardiovascular disorders. The means (± SDs) age and body weight were 8.1 (± 3.9) years and 8.8 (± 6.5) kg, respectively. The non-bulldog brachycephalic breeds included 16 (20%) Chihuahuas, 11 (13.7%) Boston Terriers, 10 (12.5%) each of Pugs, Pekingese, and Cavalier King Charles Spaniels, 4 (5%) each of Shih Tzus, Pomeranians, and Miniature Shar Pei, 3 (3.8%) Staffordshire Bull Terriers, and 2 (2.5%) each of Lhasa Apso, Bichon Frise, Brussels Griffons, and Chow Chows. Among the investigated 80 dogs, there were 44 males (29 castrated) and 36 females (33 spayed).

### Radiographic measurements

**Fig. 2:**
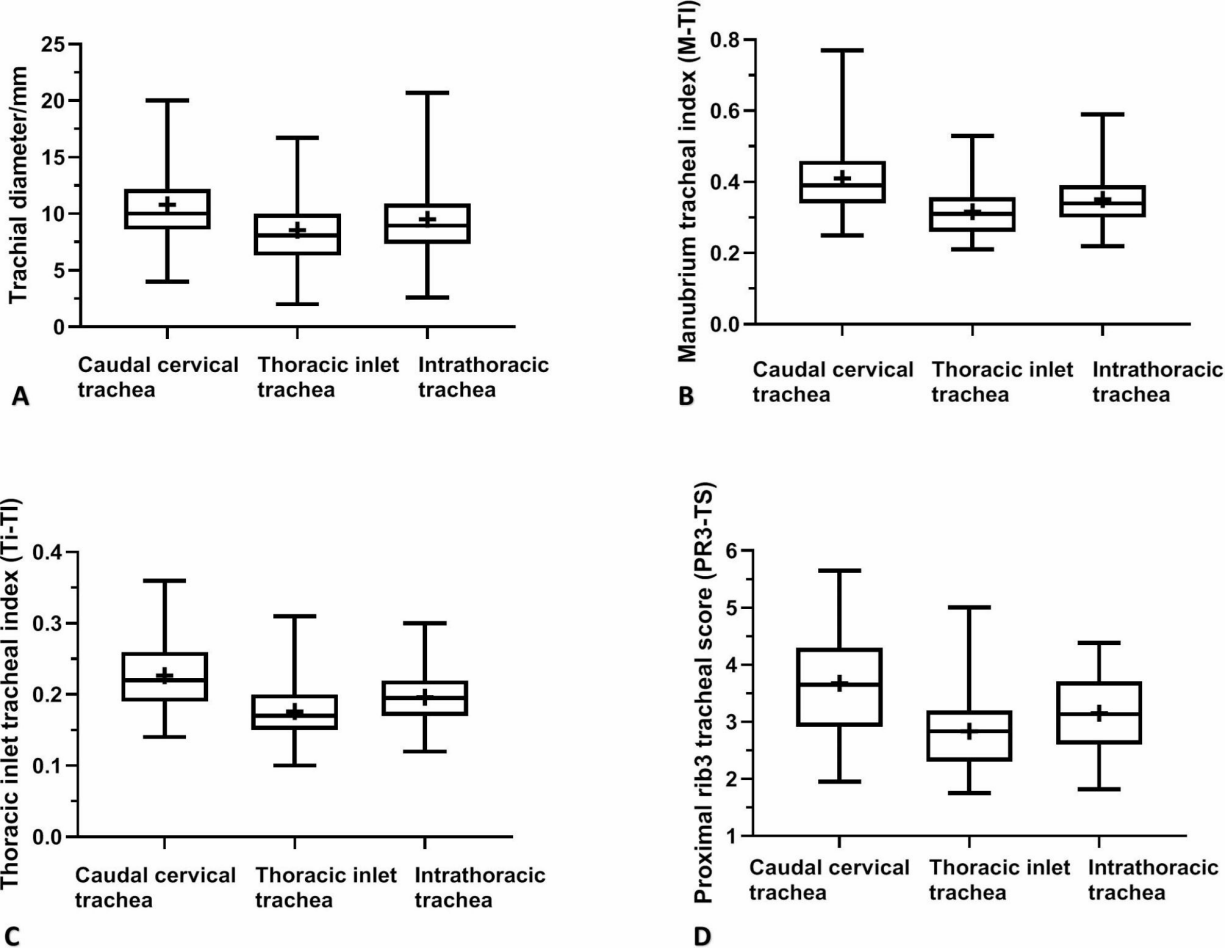
Box-and-whisker plots of vertical tracheal diameter (A), manubrium-tracheal index (B), thoracic inlet-tracheal index (C), and proximal rib3-tracheal score (D) at the caudal cervical, thoracic-inlet, and intra-thoracic tracheal regions for 80 healthy non-bulldog brachycephalic breeds. The 25th to 75th percentiles and ranges are represented by boxes and whiskers, respectively; the medians and means are represented by lines and crosses within the boxes, respectively

There were significant differences (*P* ≤ 0.0001) among the mean absolute and standardized VTDs calculated at the caudal cervical, thoracic-inlet, and intra-thoracic tracheal regions. The greatest difference was noted between the means caudal cervical and thoracic-inlet tracheal diameters, with the lowest mean VTD being identified at the thoracic-inlet region (Table 1; Fig. 2). The mean VTD calculated at the thoracic-inlet region (8.6 mm) was 20.4% and 10.9% less than those calculated at the caudal cervical (10.8 mm) and intra-thoracic (9.5 mm) tracheal regions, respectively.

**Table 1:** Means (± SDs) and 95% CIs for the radiographic measurements of the absolute, averaged, and standardized values of vertical tracheal diameter (VTD) at caudal cervical, thoracic-inlet, and intra-thoracic tracheal regions for 80 non-bulldog brachycephalic small-breed dogs without evidence of pulmonary or cardiovascular disease

Variables	Mean ± SD	95% CI		P-value < 0.05	
**Absolute vertical tracheal diameter (VTD)/mm**			**ANOVA test**	**Tukey’s test**	**Unpaired t-test**
Caudal cervical (A) VTD (mid-C5)	10.8 ± 3.2	10.1–11.6		A-B, p < 0.0001	A-B, p < 0.0001
Thoracic inlet (B) VTD (ca-C7)	8.6 ± 3.0	7.9–9.2	P = 0.0001	A-C, p = 0.036	A-C, p = 0.017
Intrathoracic (C) VTD (mid-T2-T3)	9.5 ± 3.3	8.8–10.3		B-C, p = 0.137	B-C, p = 0.057
**Averaged VTD**	**9.7 ± 3.2**	**8.9–10.4**			
**Standardizing parameter/mm**					
Manubrium-length (ML)	27.5 ± 8.4	25.6–29.3			
Thoracic inlet-distance (Ti-D)	48.6 ± 13.6	45.5–51.6			
Proximal R3-width (PR3-W)	**3.1 ± 1.1**	**2.9–3.3**			
**Manubrium-tracheal index (M-TI)**			**ANOVA test**	**Tukey’s test**	**Unpaired t-test**
M-TI (caudal cervical trachea, A)	0.41 ± 0.10	0.39–0.43		A-B, p < 0.0001	
M-TI (thoracic inlet trachea, B)	0.32 ± 0.07	0.30–0.33		A-C, p < 0.0001	
M-TI (intrathoracic trachea, C)	0.35 ± 0.07	0.34–0.37		B-C, p = 0.020	
**Averaged M-TI**	**0.36 ± 0.08**	**0.34–0.38**			
**Thoracic inlet-tracheal index (Ti-TI)**					
Ti-TI (caudal cervical trachea, A)	0.23 ± 0.05	0.22–0.24		A-B, p < 0.0001	A-B, p < 0.0001
Ti-TI (thoracic inlet trachea, B)	0.18 ± 0.04	0.17–0.18	P < 0.0001	A-C, p < 0.0001	A-C, p < 0.0001
Ti-TI (intrathoracic trachea, C)	0.20 ± 0.04	0.19–0.20		B-C, p = 0.006	B-C, p ≤ 0.003
**Averaged Ti-TI**	**0.20 ± 0.04**	**0.19–0.21**			
**Proximal R3-tracheal score (PR3-TS)**					
PR3-TS (caudal cervical trachea, A)	3.7 ± 0.9	3.5–3.9		A-B, p < 0.0001	
PR3-TS (thoracic inlet trachea, B)	2.8 ± 0.7	2.7–3.0		A-C, p < 0.0001	
PR3-TS (intrathoracic trachea, C)	3.2 ± 0.6	3.0–3.3		B-C, p = 0.018	
**Averaged PR3-TS**	**3.2 ± 0.7**	**3.1–3.4**			

**Fig. 3:**
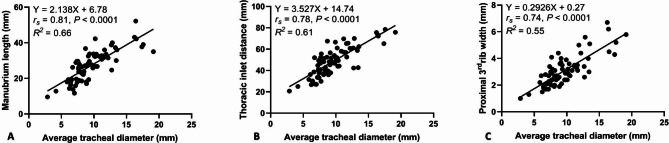
Scatter plots of the average vertical tracheal diameter (VTD) versus manubrium length (ML, A), thoracic-inlet distance (Ti-D, B), and proximal 3rd rib width (PR3-W, C) identified for 80 healthy non-bulldog brachycephalic breeds

**Fig. 4:**
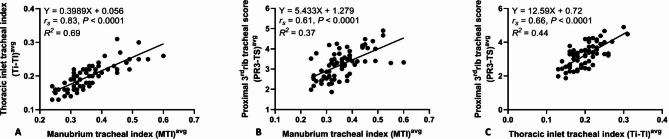
Scatter plots of the averaged manubrium-tracheal index (M-TI) versus the averaged thoracic-inlet tracheal index (Ti-TI, A) and the averaged proximal 3rd rib tracheal score (PR3-TS, B), as well as the averaged Ti-TI versus the averaged PR3-TS (C) identified for 80 healthy non-bulldog brachycephalic breeds

A better correlation existed between the average VTD and manubrium length (ML, *r*_*s*_= 0.81, *P* < 0.0001) compared to the average VTD and thoracic inlet distance (Ti-D, *r*_*s*_= 0.78, *P* < 0.0001) and the average VTD and proximal 3rd rib-width (PR3-W, *r*_*s*_= 0.74, *P* < 0.0001) (Fig. 3). There was a stronger correlation identified between the M-TI and Ti-TI (*r*_*s*_= 0.83, *P* < 0.0001) compared to the M-TI and PR3-TS techniques (*r*_*s*_= 0.61, *P* < 0.0001) (Fig. 4A **and B**). Moreover, there was a significant correlation determined between the Ti-TI and PR3-TS (*r*_*s*_= 0.66, *P* < 0.0001) (Fig. 4C).

## Discussion

The results of the current study demonstrated that in non-bulldog brachycephalic small-breed dogs, vertical tracheal diameters (VTDs) varied (*P* ≤ 0.0001) in compliance with the region of the corresponding trachea, with the highest variation being identified between the caudal cervical and thoracic-inlet VTDs. Thoracic-inlet VTD was 20.4% and 10.9% narrower compared to caudal cervical and intra-thoracic VTDs, respectively. A better correlation was noted between the average VTD and ML compared to Ti-D and PR3-W. A better correlation was determined between the M-TI and Ti-TI compared to PR3-TS. The means M-TI calculated for our healthy non-bulldog brachycephalic breeds were 0.41 at caudal cervical, 0.32 at thoracic-inlet, and 0.35 at intra-thoracic tracheal regions.

A recent similar study was performed on non-brachycephalic small-breed dogs by the same investigators [15]. In this recent study, the thoracic-inlet VTD was also 20.9% and 10.9% narrower than VTDs measured in the caudal cervical and intra-thoracic regions, respectively [15]. Similarly, a strong correlation was identified between the average VTD and ML (*rs* = 0.82 in non-brachycephalic breeds versus *rs* = 0.81 in non-bulldog brachycephalic breeds). In our previous non-brachycephalic study, there was also a stronger correlation between M-TI and Ti-TI (*rs* = 0.77) compared to that identified between M-TI and PR3-TS (*rs* = 0.63) [15]. The mean M-TI calculated at the caudal cervical, thoracic-inlet, and intra-thoracic regions were relatively higher (0.45, 0.35, and 0.39, respectively) in non-brachycephalic breeds [15] compared to those (0.41, 0.32, and 0.35, respectively) calculated for non-bulldog brachycephalic breeds in the present study.

Numerous radiographic and computed tomographic techniques have been established to diagnose congenital and acquired tracheal narrowing in dogs [3–9]. However, radiographic evaluation of tracheal size in dogs underestimated tracheal luminal diameter by approximately 1.0 mm compared to computed tomography [7]. Nonetheless, radiography is still the most widely utilized imaging modality in veterinary practice to assess VTD in dogs [10, 11]. The demand for radiographic evaluation of VTD was the necessity for diagnosing tracheal hypoplasia and for selecting the appropriate sizes of endotracheal tubes [7, 10, 11]. The routinely established Ti-TI and PR3-TS procedures relied on standardizing VTD with thoracic inlet distance (Ti-D) and proximal 3rd rib width (PR3-W), respectively [3, 5, 6, 12–14]. The widths of the proximal pair of third ribs (PR3-Ws) appeared most likely unequal on the lateral radiographic view. This inequality could be attributed to the possibility of minimal tilting of the dog during radiographic positioning [3, 5, 18] which may have biased the PR3-TS procedure. In addition, utilizing a too-small PR3-W to calculate PR3-TS was found to be more prone to errors compared to using thoracic-inlet distance to calculate (Ti-TS) [5]. Another limitation added to the PR3-TS includes the inability to measure the PR3-W in dogs with thoracic vertebral anomaly and crowded ribs, or due to the superimposition of the 3rd pair of ribs. Similarly, measuring Ti-D should be affected by the existence of thoracic vertebral anomaly and the variable landmarks previously reported to outline Ti-D. Thus, the points outlining the minimum distance of the thoracic inlet (Ti-D) were measured between the cranioventral margin of T1 and the highest level of the cranial portion of the manubrium. Nevertheless, thoracic vertebral anomaly associated with brachycephalic breeds may affect the measurement of Ti-D and subsequently the results of the Ti-TI procedure.

Therefore, our previous recent [15] and present studies utilized for the first time the length of the manubrium (ML) to standardize VTD via calculating the M-TI at the caudal cervical, thoracic-inlet, and intra-thoracic trachea in non-brachycephalic and non-bulldog brachycephalic small-breed dogs. In non-bulldog brachycephalic small-breed dogs (investigated in the current study), the mean tracheal luminal diameter measured at the thoracic-inlet region (8.6 mm) was 20.4% and 10.9% lower than those measured at the caudal cervical (10.8 mm) and intra-thoracic (9.5 mm) regions, respectively. Similar values were interestingly identified in non-brachycephalic small-breed dogs, as the mean thoracic-inlet tracheal diameter (10.6 mm) was 20.9% and 10.9% narrower compared to the means caudal cervical (13.4 mm) and intra-thoracic (11.9 mm) VTDs, respectively [15]. These percentages were found to be higher than those determined for large-breed dogs, in which the mean tracheal diameter measured at the thoracic-inlet (15.3 mm) was 5.7% and 7.6% lower than those measured at the caudal cervical (16.2 mm) and intra-thoracic (16.5 mm) regions, respectively [19]. Thus, compared to large-breed dogs, non-brachycephalic and non-bulldog brachycephalic small-breed dogs have a relatively narrower VTD at the thoracic-inlet region. Our results are in agreement with a previous report where the diameter and thickness of the tracheal rings were narrowest in the thoracic inlet region [19]. The narrowest VTD identified at the level of the thoracic inlet in our and previous studies [15, 19] is attributed to the change of the direction of the trachea at a relatively small thoracic inlet surrounded by bones [19]. Moreover, the thoracic-inlet trachea is compressed by the esophagus altering its diameter and possibly predisposing it to tracheal collapse in the thoracic-inlet region [19–22]. At the level of the thoracic-inlet region, thoracic inlet-tracheal indices (Ti-TIs) determined for healthy small-breed dogs were previously reported to be 0.11, 0.12, or 0.13 in bulldogs, 0.16 in non-bulldog brachycephalic breeds, and 0.20 or 0.21 in non-brachycephalic breeds [3, 13, 14, 23, 24]. The mean Ti-TI value (0.16) previously established at the thoracic inlet of non-bulldog brachycephalic dogs is in agreement with that (0.18) calculated in our population. The limited disparity between studies could be attributed to the limited variations in the measuring procedures of the thoracic inlet distance and the relatively bigger sample size of healthy non-bulldog brachycephalic breeds enrolled in our study. Our study considered that non-bulldog brachycephalic breeds with a Ti-TI value < 0.17 would have tracheal hypoplasia. Thus, the present study would suggest using the reported Ti-TI reference value (0.17) to radiographically distinguish tracheal hypoplasia in non-bulldog brachycephalic breeds.

In previous studies, the normal proximal 3rd rib tracheal score (PR3-TS) in the intra-thoracic trachea was higher than 3.0 [14, 25, 26]. This score is similar to the mean PR3-TS value (3.2) identified at the level of the intra-thoracic trachea in the present study. Thus, an intra-thoracic tracheal lumen with a PR3-TS value below 3.0 could be hypoplastic. However, in a different recent study, brachycephalic dog breeds with a PR3-TS less than 2.0 were suggested to have hypoplastic trachea [13]. To the best of our knowledge, radiographic determination of the caudal cervical tracheal index was not established in dogs using different standardizing parameters (ML, Ti-D, and PR3-W). Therefore, the mean values of M-TI, Ti-TI, and PR3-TS identified at the level of the caudal cervical tracheal region were, respectively, 0.41, 0.23, and 3.7 in non-bulldog brachycephalic breeds without evidence of pulmonary or cardiovascular disease.

The previously reported questionable diagnostic value associated with Ti-TI and PR3-TS [6] may propose the usefulness of utilizing M-TI to assess the three VTDs along the canine trachea. Moreover, the strong positive correlations determined in the present study between the VTD versus ML (*rs* = 0.81), and the M-TI versus Ti-TI (*rs* = 0.83) would support the usefulness of utilizing M-TI as an alternative simple procedure to evaluate VTD in non-bulldog brachycephalic breeds. Nevertheless, the present study did not calculate the inter- and intra-investigator repeatability of the M-TI, despite the expected reliability of such a procedure as a result of the simplicity of measuring the ML and the corresponding VTD. Another study limitation is the sole assessment of tracheal diameter in healthy subjects; thus, further investigation should be achieved to validate the M-TI procedure in healthy versus dyspneic non-brachycephalic and brachycephalic dogs. Testing the difference in the VTDs among non-brachycephalic, non-bulldog brachycephalic, and bulldog breeds is another future study recommended by the authors.

## Conclusions

The absolute and standardized tracheal diameters calculated along the tracheal lumen varied with the region of the trachea, with the narrowest lumen being noted at the level of the thoracic-inlet region. M-TI may be a proper alternative to the conventional Ti-TI and PR3-TS for radiographic evaluation of tracheal diameter in dogs. Averaged M-TI, Ti-TI, or PR3-TS < 0.34, < 0.19, or < 3.1, respectively, may designate hypoplastic trachea in non-bulldog brachycephalic breeds. The current study proposes a future screening program (i.e. VTD scheme) for detection of hypoplastic trachea in dogs using the M-TI. However, future validation of the M-TI in healthy and dyspneic dogs is still warranted.

## Data Availability

Data supporting the study results are included in the article. Row data are available upon request to AM (aymostafa@ cu.edu.eg). The dataset is publicly available in the following web link:-. Tracheal Index_Nonbulldog Brachy.xlsx.

## Abbreviations

VTD: Vertical tracheal diameter
ML: Manubrium length
Ti-D: Thoracic-inlet distance
PR3-W: Proximal 3rd rib width
M-TI: Manubrium-tracheal index
Ti-TI: Thoracic-inlet tracheal index
PR3-TS: Proximal 3rd rib tracheal score
